# Identifying Key Markers for Monofloral (Eucalyptus, Rosemary, and Orange Blossom) and Multifloral Honey Differentiation in the Spanish Market by UHPLC-Q-Orbitrap-High-Resolution Mass Spectrometry Fingerprinting and Chemometrics

**DOI:** 10.3390/foods13172755

**Published:** 2024-08-29

**Authors:** Araceli Rivera-Pérez, Alba María Navarro-Herrera, Antonia Garrido Frenich

**Affiliations:** Research Group “Analytical Chemistry of Contaminants”, Department of Chemistry and Physics, Research Centre for Mediterranean Intensive Agrosystems and Agrifood Biotechnology (CIAIMBITAL), Agrifood Campus of International Excellence (ceiA3), University of Almeria, E-04120 Almeria, Spain; arp800@ual.es (A.R.-P.); am.navarroherrera@gmail.com (A.M.N.-H.)

**Keywords:** honey, food authentication, botanical origin, untargeted analysis, high-resolution mass spectrometry, metabolomics, foodomics, fingerprinting, multivariate data analysis, markers

## Abstract

Honey differentiation based on the botanical origin is crucial to guarantee product authenticity, especially considering the increasing number of fraud cases. This study assessed the metabolomic differences arising from various botanical origins in honey products sold in Spanish markets, focusing on two goals: (1) discrimination within monofloral samples (eucalyptus, rosemary, and orange blossom honey) and (2) differentiation between multifloral vs. monofloral honey samples. An omics strategy based on ultra-high-performance liquid chromatography coupled with quadrupole-Orbitrap-high-resolution mass spectrometry (UHPLC-Q-Orbitrap-HRMS) was applied for the reliable identification of specific honey markers selected by orthogonal partial least squares discriminant analysis (OPLS-DA) (R^2^Y = 0.929–0.981 and Q^2^ = 0.868–0.952), followed by the variable importance in projection (VIP) approach. Key amino acid, alkaloid, and trisaccharide markers were identified to distinguish between honey samples. Some Amadori compounds were highlighted as eucalyptus honey markers, suggesting their potential use for honey aging and botanical origin differentiation. L-phenylalanine and raffinose were markers of rosemary honey. Four markers (e.g., trigonelline, L-isoleucine, and *N*-(1-deoxy-1-fructosyl)isoleucine) were found in higher levels in multifloral samples, indicating a greater availability of amino acids, potentially increasing the Maillard reaction. This research is the first to address the botanical origin’s impact on honey by identifying novel markers not previously described.

## 1. Introduction

Honey, a natural sweet substance produced by honeybees from the nectar of flowers, has been valued for its nutritional and medicinal properties since ancient times [1]. Its composition, which includes bioactive substances, such as sugars, amino acids, vitamins, minerals, flavonoids, organic acids, and Maillard reaction products [2,3], is influenced by the botanical origin, geographical location, and environmental conditions (among other factors), which significantly impact its organoleptic and overall properties [4]. However, the authenticity and quality of honey has become an important object of study because of the increasing prevalence of adulteration and mislabeling practices in the market [5], being honey the third-most vulnerable food product according to the 2023 Food Authenticity Network reports [6].

Among all the influential factors, harvest conditions (e.g., botanical origin) play a key role in determining the fingerprint of honey [7]. The differentiation of honey based on the botanical origin is essential for ensuring product authenticity and consumer safety. In this context, two types of honey may be distinguished based on their botanical origin: multifloral and monofloral honey [8,9]. Monofloral honey is mainly derived from one flower type, offering a unique flavor. Multifloral honey comes from multiple flower nectars, leading to a more complex and varied profile. In Spanish cuisine, monofloral honey, such as that from eucalyptus, rosemary, or orange blossom, is highly prized and widely demanded for its distinct flavors and health benefits.

Advanced analytical techniques, such as metabolomics, along with multivariate statistical methods, have emerged as powerful tools for monitoring honey quality, including nuclear magnetic resonance [10,11] and elemental fingerprinting [12,13]. In recent decades, ultra-high-performance liquid chromatography (UHPLC), an advanced version of LC, has gained significant attention in food authentication due to its higher resolution, reduced analysis times, and enhanced retention time reproducibility [14]. Additionally, combining LC with high-resolution mass spectrometry (HRMS) allows untargeted omics approaches, such as fingerprinting, by offering high sensitivity, accurate mass measurement, and structural elucidation for reliable metabolite identification [15], enabling the identification of specific markers that can verify honey’s botanical origin.

LC-HRMS-based omics—using Orbitrap or quadrupole-time-of-flight (QTOF) analyzers—has been applied using phenolic profiling approaches combined with partial least squares discriminant analysis (PLS-DA) for honey authentication based on various botanical and geographical origins [9], as well as for Greek vs. Polish honey differentiation [16]. LC-HRMS fingerprinting and PLS-DA discriminated between honey produced in Spain, such as eucalyptus, rosemary, and thyme varieties, identifying their fingerprints as chemical descriptors for honey classification [17]. Similarly, UHPLC-HRMS honey fingerprints combined with orthogonal PLS-DA (OPLS-DA) have been exploited for the quality control of *Citrus* honey produced in case study areas of Egypt, Italy, and Greece [18]. Li et al. [19] explored the feasibility of UHPLC-Q-Orbitrap-HRMS analysis to identify litchi and acacia honey geographical markers, including the structural elucidation of key markers by MS and MS/MS data, but the chemical identity was not provided for each marker. Nowadays, the application of ambient mass spectrometry, including direct analysis in real-time mass spectrometry (DART-MS), which is simple and requires minimal sample preparation [20], is continuously increasing in honey authentication applications, such as the differentiation of monofloral chestnut and acacia samples from different geographical origins [21]. Other novel approaches, such as data fusion omics combining NMR and LC with Orbitrap and QTOF analyzers, were used for the discrimination of honey from monofloral origins (acacia, orange blossom, lavender, and eucalyptus) and multifloral sources from various geographical origins, but only three potential markers were highlighted, and they were not further chemically identified by using the available literature or databases [22]. Hence, the state-of-the-art approaches revealed the importance of searching for markers that define honey’s botanical origin, being a task not resolved yet, although it has been the subject of research in recent years.

In this work, UHPLC-Q-Orbitrap-HRMS fingerprinting and multivariate data analysis is presented as a reliable tool to differentiate botanical origins (eucalyptus, rosemary, orange blossom, and multifloral honey) in honey products sold in Spanish markets, enabling for the first time the identification of nine different specific markers that contribute to improved traceability and authenticity verification in the honey industry.

## 2. Materials and Methods

### 2.1. Honey Samples

A total of 40 different honey samples were considered in this study. First, the variation in the honey fingerprint based on the botanical origin was assessed by considering three honey types widely used in Spanish cuisine: n = 10 eucalyptus honey samples, n = 10 rosemary honey samples, and n = 10 orange blossom honey samples. Second, metabolomic differences between monofloral and multifloral honey were investigated by comparing these aforementioned honey samples, with n = 10 multifloral honey samples. All the samples were purchased in local supermarkets (Almeria, Spain), and they represented a wide range of honey varieties that are commercially available in supermarket chains throughout Spain. Table S1 displays the details of the 40 honey samples. All the samples were stored in their original packaging and maintained at 2.5 °C in darkness until further UHPLC-Q-Orbitrap-HRMS analysis.

### 2.2. Sample Preparation and Extraction

Honey samples were homogenized and tempered at room temperature for 1 h before their extraction. Honey fingerprints were obtained using a multi-sample rotary agitation method, with a subsequent clean-up step based on dispersive solid-phase extraction (dSPE).

First, 1.00 ± 0.01 g of the honey sample was weighed into a 50 mL Falcon tube using a PB1502-S/FACT analytical balance (Mettler Toledo, Barcelona, Spain). Next, 50 µL of a chlorantraniliprole solution (200 mg/kg in acetonitrile, LC-MS grade; Carlo Erba Reagents, Barcelona, Spain) was added as an internal standard to ensure optimum extraction process performance, resulting in a final concentration of 1000 µg/kg of chlorantraniliprole in the raw honey extract. Chlorantraniliprole (97.2% purity; Dr. Ehrenstorfer, Augsburg, Germany) was chosen as the surrogate standard due to its stability and absence in honey. Subsequently, 10 mL of a methanol (MeOH, LC-MS grade, Carlo Erba Reagents) and water (LC-MS grade, PanReac, Barcelona, Spain) solution (80:20, *v*/*v*) was added. The tube was vortexed for 10 s and shaken for 25 min at room temperature using a Heidolph Reax 2 rotatory agitator (Dicsa, Almeria, Spain). After agitation, raw honey extracts were cleaned up by dSPE using 50 mg of primary secondary amine (PSA) and 150 mg of MgSO_4_ per mL. The honey extracts were then vortexed for 10 s and rotatory-shaken for 5 min at room temperature. The honey extracts were centrifuged at 7500 rpm (8170× *g*) for 5 min using a Frontier™ 5816 centrifuge (Ohaus^®^, Nänikon, Switzerland), and the supernatants were filtered with 0.2 µm nylon filters. Finally, the honey extracts were diluted 10-fold using MeOH–water (80:20, *v*/*v*) and transferred to vials for further analysis. According to the whole extraction method, the honey samples were diluted 100-fold before their injection into the UHPLC-HRMS system.

All the honey samples were randomly extracted in triplicate and randomly analyzed to minimize analytical bias. Procedure blanks and quality control (QC) samples were injected at the start, after every 15 samples, and at the end of the analysis batch. Two QC samples were prepared considering the two goals of this study by mixing equal aliquots of each honey extract. For experiment 1 (i.e., discrimination between eucalyptus, rosemary, and orange blossom monofloral honey), 50 µL of honey extracts from these three types were mixed for the QC sample. For experiment 2 (discrimination between monofloral vs. multifloral honey), the QC sample was prepared by mixing 50 µL of each eucalyptus, rosemary, and orange blossom (denoted as monofloral ones) and multifloral honey extract.

### 2.3. Honey Fingerprinting by UHPLC-Q-Orbitrap-HRMS Untargeted Analysis

Honey fingerprinting was carried out using a Vanquish LC chromatograph (Thermo Fisher Scientific, Waltham, MA, USA) comprising a binary pump, a degasser, a temperature-controlled column compartment, and an autosampler. Chromatographic separation was performed using an ACE Excel 2 C18-PFP column (100 mm × 2.1 mm i.d., 2.0 µm particle size; Symta, Madrid, Spain). The mobile phase comprised ultrapure water with 0.1% formic acid (phase A) and 100% methanol (phase B). The gradient elution program started with 95% A for 2 min, followed by a decrease to 0% A over 6 min, which was maintained for 9 min. A re-equilibration step then returned the conditions to 95% A in 1 min and held for 2 min. The entire run time was 20 min per sample. The mobile phase flow rate was constant at 0.25 mL/min, and 5 µL of each sample was injected into the UHPLC-HRMS system. The column compartment was maintained at 30 °C.

The Q-Exactive™ hybrid quadrupole-Orbitrap high-resolution mass spectrometer (Thermo Fisher Scientific) operated in both positive and negative electrospray ionization (ESI) modes, considering the following parameters: a spray voltage of 4 kV, nitrogen as the sheath gas (flow rate of 35) and auxiliary gas (flow rate of 10), the s-lens RF level at 50, the auxiliary gas heater temperature at 300 °C, and the capillary temperature at 300 °C. Full-scan mode was used for HRMS data acquisition of precursor ions in the mass-to-charge ratio (*m*/*z*) range of 70–1000 for both ESI+ and ESI− modes, with a maximum injection time (IT) of 256 ms, an automatic gain control (AGC) target of 1 × 10^6^, and a resolution of 70,000 FWHM (*m*/*z* 200). MS/MS data acquisition (acquisition of fragment ion data) was performed using the data-dependent dd-MS^2^ (Top 5) mode within the *m*/*z* 70–1000 range in both ESI+ and ESI− modes. This mode involved the higher-energy collisional dissociation (HCD) collision cell with normalized collision energies (NCEs) of 20, 35, and 55 eV. MS/MS data were acquired with a maximum IT of 64 ms, an isolation window of 2 *m*/*z*, an AGC target of 1 × 10^5^, a dynamic exclusion time of 10 s, and a resolution of 17,500 FWHM at *m*/*z* 200 for both polarities.

### 2.4. Processing Honey Omics Data: Designed Untargeted Metabolomics Workflow Using Compound Discoverer^TM^ Software

A metabolomics workflow was developed for the untargeted analysis of honey fingerprints using Compound Discoverer™ version 3.3 software (Thermo Fisher Scientific). Initially, honey fingerprint data (.raw files) were imported into the software. For the first experiment (differentiation between monofloral honey samples), three sample groups were established, namely eucalyptus, rosemary, and orange blossom varieties, and thus, 90 raw files were considered. In the second experiment (discrimination between monofloral and multifloral honey), two sample groups were indicated, monofloral (including eucalyptus, rosemary, and orange blossom honey samples, n = 90) and multifloral (n = 30), leading to a total of 120 raw files to be processed. Furthermore, procedure blanks and quality control (QC) samples were included in the workflow for background signal removal and area refinement. Procedure blanks were categorized as “blank” and QC samples as “quality control”.

First, feature extraction was carried out within the retention time (RT) and *m*/*z* ranges of 0–20 min and 70–1000, respectively, for both ESI+ and ESI− ionization modes. Further extraction parameters included a mass tolerance of 5 ppm, as well as a minimum peak intensity of 1 × 10^5^. Next, features were aligned within honey samples, considering a QC raw file using the ChromAlign algorithm [23], and grouped with mass and RT tolerances of 5 ppm and 0.2 min, respectively. A mass tolerance of 5 ppm and an S/N threshold of 1.5 were set for gap filling.

Subsequently, feature area refinement was performed by applying QC correction, using a QC coverage threshold of 60% (features must be detected in at least 60% of QC samples to be annotated), and adjusting the maximum relative standard deviation (RSD) of the feature area in QCs from an initial 25% to a final 20%. Background filtering was carried out by setting the maximum allowed ratio of sample to blank at 5 for consideration as a blank feature. The data were then normalized by the median (normalized to the maximum peak area median across all the samples).

Compound identification was performed by matching to LC-MS spectral libraries, including ChemSpider, BioCyc, ChEBI, ChemBank, FDA, FooDB, HMDB, KEGG, LIPID MAPS, MassBank, MolBank, Phenol-Explorer, PlantCyc, PubMed, mzCloud, and mzVault, with a mass tolerance of 3 ppm for precise annotations. The workflow also included differential analysis between eucalyptus, rosemary, and orange blossom groups, providing *p*-values from one-way ANOVA with Tukey’s post-hoc test. Two-tailed Student’s *t*-test was used for multifloral vs. monofloral comparison. In all the cases, *p*-values were Benjamini–Hochberg-adjusted for the false discovery rate. The workflow also provided fold change analysis expressed as log2(FC) values. Finally, the normalized peak area data matrix was exported in .csv format for further multivariate data analysis.

### 2.5. Multivariate Data Analysis

SIMCA^®^ version 17 software (Umetrics, Umeå, Sweden) was used for unsupervised and supervised multivariate data analysis considering the normalized data matrix (.csv) obtained from the untargeted analysis. Initially, the data were Pareto-scaled using SIMCA^®^ software, and no further data treatment was applied.

The natural clustering trend among honey samples was assessed by unsupervised principal component analysis (PCA) and hierarchical cluster analysis (HCA, Ward’s linkage method) considering all the samples, which was also used to identify potential outliers within honey samples. For experiment 1 (discrimination between eucalyptus, rosemary, and orange blossom monofloral honey), the dataset for unsupervised analysis included 103 observations (90 honey samples and 13 QC samples). In experiment 2 (multifloral vs. monofloral honey differentiation), 133 observations were taken into account (120 samples and 13 QC samples). Notably, QC samples were only included in the unsupervised models.

For supervised OPLS-DA modeling, which was further used for marker identification, the dataset was divided into a training set (80% of the total observations), used for model building, and a prediction set (20% of the total), which was reserved for external validation. Identification and selection of markers were performed by considering four OPLS-DA models made of a pair of conditions for tested botanical origins: rosemary vs. eucalyptus honey, rosemary vs. orange blossom honey, and orange blossom vs. eucalyptus honey (experiment 1) and multifloral vs. monofloral honey (experiment 2).

The good performance of the OPLS-DA models was evaluated using the goodness-of-fit (R^2^Y) and goodness-of-prediction (Q^2^) metrics, with a Q^2^ cut-off of >0.5 indicating good predictability. Additionally, the models successfully overcame internal validation when a CV-ANOVA *p*-value of <0.05 (indicating model significance) was obtained through SIMCA^®^ default seven-fold cross-validation (CV). Permutation tests (n = 200 permutations) were also considered for each sample class to discard model overfitting. External validation was performed by assessing the correct classification rate (CCR%) for the prediction set samples.

### 2.6. Workflow to Identify Honey Markers

The variable importance in projection (VIP) tool from SIMCA^®^ software was used to identify the most significant variables (compounds) for distinguishing between honey groups. For that, variables from the corresponding OPLS-DA model with a VIP score of >1.00 were studied. Moreover, additional restrictions were applied for reliable markers, and thus, these variables had to represent significant differences among sample groups (Benjamini–Hochberg-adjusted *p*-values < 0.05) to be further examined as potential markers. Following this, fold change (FC) analysis was conducted with a cut-off value of FC > 1.10, expressed as log2(FC) values, to compare rosemary vs. eucalyptus honey, rosemary vs. orange blossom honey, and orange blossom vs. eucalyptus honey (experiment 1) and multifloral vs. monofloral honey (experiment 2), highlighting accumulation trends (up-/down-accumulation) of markers. Boxplots generated by Compound Discoverer^TM^ software were used to visualize the relative contents of markers within sample groups.

The MetaboAnalyst 6.0 platform (https://www.metaboanalyst.ca/ (accessed on 25 July 2024)) was used to create heatmaps (Euclidean distance, Ward clustering algorithm) of selected VIP markers and create ROC curves for each proposed marker. IBM SPSS Statistics version 25 software (Armonk, NY, USA) was used to generate a control chart to verify extraction performance, considering the chromatographic peak areas of chlorantraniliprole.

For reliable marker identification, LC-HRMS parameters included two ions (precursor and at least one fragment ion) with mass accuracy ≤ 5 ppm per metabolite, with fully overlapping chromatographic peaks at the metabolite retention time. These MS and MS/MS ions were automatically matched with LC-MS libraries in the Compound Discoverer^TM^ untargeted workflow. To avoid false positives, a thorough manual inspection of fragment ions was conducted. Theoretical MS/MS fragments from each marker were elucidated using in silico fragmentation tools, such as Mass Frontier^TM^ version 7.0 software (Thermo Fisher Scientific), Competitive Fragmentation Modeling for Metabolite Identification (CFM-ID, https://cfmid.wishartlab.com/ (accessed on 25 July 2024) [24]), MetFrag (https://ipb-halle.github.io/MetFrag/ (accessed on 25 July 2024) [25]), and MS/MS databases, like mzCloud (https://www.mzcloud.org/ (accessed on 25 July 2024)). Consequently, identification confidence level 2 (putatively annotated compounds or probable structures) was achieved for all marker compounds following established HRMS-based identification confidence levels [26,27].

A summary of the criteria that model variables must fulfill to be considered as potential markers is presented, including statistical and UHPLC-HRMS identification requirements:

- VIP score cut-off > 1.00;
- Benjamini–Hochberg-adjusted *p*-values < 0.05;
- FC cut-off > 1.10;
- Two characteristic ions (precursor and at least one fragment ion) with mass accuracy ≤ 5 ppm;
- Fully overlapping chromatographic peaks of the ions at the metabolite retention time (RT ± 0.10 min);
- Study of the potential logical occurrence of the proposed marker in the study matrix based on the relevant published literature and food databases (if available).

## 3. Results and Discussion

### 3.1. Unsupervised Multivariate Data Analysis

#### 3.1.1. Overview of Monofloral (Eucalyptus, Rosemary, and Orange Blossom) Honey

Untargeted UHPLC-Q-Orbitrap-HRMS metabolomics was used to examine how the botanical origin affects the composition of honey, reflecting the different floral sources from which bees collected nectar. Honey products that are labeled as “monofloral” indicate that the honey is predominantly derived from the nectar of a single type of flower. This classification is based on the supplier information and can often be found in honey varieties in supermarkets.

In this context, first, three widely used honey varieties labeled as “monofloral” were compared: eucalyptus, rosemary, and orange blossom honey. A total of 162 features were identified using the untargeted metabolomics approach from monofloral honey fingerprints, with 94 of them having available experimental MS/MS data, which were then analyzed using multivariate statistical methods to identify key markers.

For illustration, Figure S1 displays representative total ion chromatograms (TICs) of monofloral honey samples obtained through full-scan-mode acquisition in both positive and negative polarities (ESI+ and ESI−) using untargeted UHPLC-Q-Orbitrap-HRMS analysis. A visual inspection of ESI+ and ESI− TICs indicated that despite the different botanical origins of the honey samples, metabolomic differences due to floral sources were not immediately obvious, indicating the need for further advanced statistical tools to extract discriminant information from recorded honey omics data. In more detail, TIC inspection would reveal more differences in the ESI+ profile within monofloral samples compared to variations between negative ionized (ESI−) metabolites. Moreover, TICs of both polarities revealed the predominance of highly polar compounds (e.g., amino acids and sugars) that appeared at the front of the chromatograms in reverse-phase LC (Figure S1).

**Figure 1:**
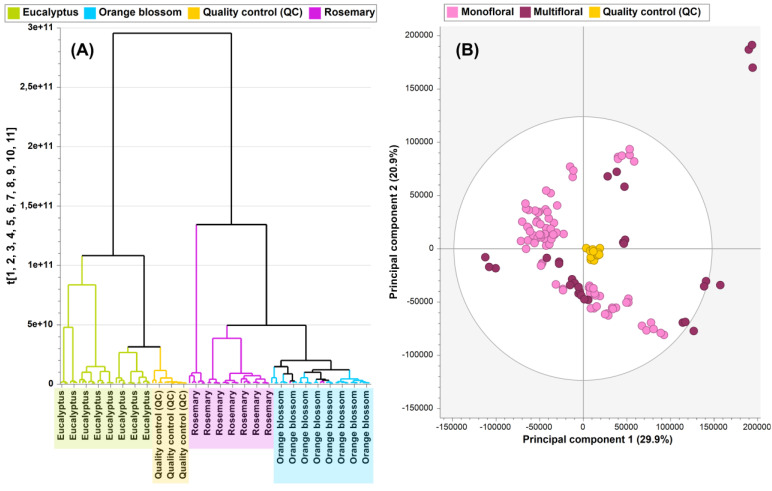
(**A**) Dendrogram obtained by HCA for monofloral samples (eucalyptus, rosemary, and orange blossom honey). (**B**) PCA score plot (PC1 vs. PC2) of multifloral vs. monofloral (eucalyptus, rosemary, and orange blossom) honey.

Once chromatograms were inspected, an unsupervised HCA was built, considering all monofloral samples (i.e., 30 samples of eucalyptus, rosemary, and orange blossom honey analyzed in triplicate, leading to a total of 90 observations) to assess the variance among samples related to the botanical origin. Moreover, 13 QC samples acquired within the analytical run were considered in this unsupervised step to check the good performance of the instrumental and untargeted analysis. Thus, HCA was performed considering 103 observations and 162 variables (honey features). The resulting HCA (Figure 1A) revealed four main clusters corresponding to the three types of investigated honey, as well as a tight cluster for QC samples. The tight QC cluster displayed the excellent performance and robustness of the instrumental and chemometrics analysis. Interestingly, HCA is also postulated as an unsupervised tool to highlight potential sample outliers. In this regard, one rosemary sample (“Helios” brand, Table S1) was found by HCA as an outlier that mainly clustered apart from rosemary-type samples, and it was associated with the orange blossom metabolomic profile (Figure 1A). In line with these findings, the “Helios” rosemary sample presented a slightly different appearance compared to the remaining ones, since it was characterized by a more viscous texture. Furthermore, the HCA findings revealed higher similarities between the metabolomic profiles of rosemary and orange blossom honey in comparison with eucalyptus-type samples, which formed an individual cluster. According to these outcomes, this rosemary sample (and their three replicates) was no longer considered in the statistical analysis.

#### 3.1.2. Overview of Multifloral vs. Monofloral Honey

Multifloral honey is derived from the nectar of multiple types of flowers. Whereas monofloral honey primarily comes from one floral source, multifloral honey is produced when bees collect nectar from various flower species. Multifloral honey is often more common and generally available, reflecting the diverse ecosystems from which it is sourced.

Thus, the second goal of this study was to assess metabolomic differences between multifloral and monofloral honey. The proposed untargeted metabolomics approach identified 200 features from multifloral and monofloral honey fingerprints, with 112 of them having available experimental MS/MS data. Similar to the trends found for monofloral honey samples, the visual inspection of ESI+ and ESI− TICs showed that metabolomic differences between sample groups are challenging to appreciate at first sight (Figure S1).

After inspecting the chromatograms, PCA was performed considering previous monofloral samples (n = 90) and 10 multifloral honey samples analyzed in triplicate (n = 30), including QC samples (n = 13), as well as 200 variables (honey features). A bidimensional score plot was created using the first two PCs, with PC1 explaining 29.9% of the variability and PC2 accounting for 20.9% of the variance (Figure 1B). PCA did not distinctly separate multifloral and monofloral samples. Consequently, these challenging metabolomic differences would require further supervised statistical modeling. The observed variability within the honey samples could be attributed to important differences in their chemical composition, reflecting the wide botanical origin range and nectar types, especially within multifloral samples (Figure 1B). In more detail, “Miel de Alcarria” (located in the upper-right region outside the confidence ellipse) notably clustered apart from the remaining multifloral samples (Figure 1B), most likely due to its protected designation of origin (PDO) condition resulting in a distinguished type of honey from the Alcarria region in Spain, recognized for its unique and high-quality characteristics. Another multifloral sample (“Didilo”) clustered apart (in the left region of the PCA plot, with negative PC1 loadings) from most of the multifloral samples that were widespread within the PCA score plot. The resulting PCA model comprised 14 principal components (PCs), accounting for 90.3% of the total variance (R^2^X = 0.903) and, in line with the clustering overview, showed a cross-validation coefficient higher than 0.5 but not close to 1 (Q^2^ = 0.746).

As described, the quality of the instrumental analysis was demonstrated using QC samples by obtaining tight QC clusters (Figure 1). Additionally, the good performance of sample preparation and extraction of honey samples was verified by monitoring the chromatographic peak area of the surrogate internal standard (chlorantraniliprole; RT = 10.50 ± 0.10 min, precursor ion *m*/*z* 483.97602) within honey fingerprints. A control chart of these areas (Figure S2) showed that all samples were within the upper and lower control limits (±3 × standard deviation, σ), with no outliers detected during the honey extraction process. All these quality control activities guaranteed that the whole metabolomics workflow was under control.

### 3.2. Supervised Multivariate Data Analysis

#### 3.2.1. Experiment 1: OPLS-DA to Discriminate Monofloral Samples (Eucalyptus, Rosemary, and Orange Blossom Honey)

Three OPLS-DA models were developed to differentiate monofloral samples based on their botanical origin and search for key specific markers by using the following pairs of conditions: rosemary vs. eucalyptus honey, rosemary vs. orange blossom honey, and orange blossom vs. eucalyptus honey. The models were constructed using 80% of the total observations (i.e., 48 observations) as the training set, while the remaining 20% (i.e., 12 observations) were randomly selected for external validation. Internal validation of the OPLS-DA models was performed using SIMCA^®^ seven-fold CV, achieving significance with a CV-ANOVA *p*-value of <0.05, and the models were further validated through permutation tests (200 permutations) to discard model overfitting. External validation was assessed considering the misclassification table and the CCR% for blindly predicted samples from the prediction set. Detailed validation and performance parameters are presented in Table S2.

**Figure 2:**
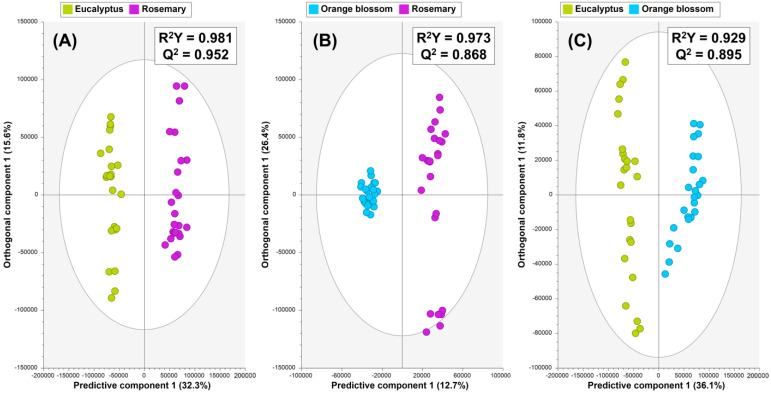
OPLS-DA score plots made of 80% of total honey fingerprints (training sets) showing the following discrimination of samples: (**A**) rosemary vs. eucalyptus honey, (**B**) rosemary vs. orange blossom honey, and (**C**) orange blossom vs. eucalyptus honey.

The OPLS-DA models (Figure 2), based on 80% of UHPLC-Q-Orbitrap-HRMS honey fingerprints (training sets), demonstrated robust internal CV parameters, with high goodness of fit (R^2^Y = 0.929–0.981) and predictive ability (Q^2^ = 0.868–0.952), and CV-ANOVA *p*-values < 0.05 (Table S2). Q^2^ values > 0.9 supported the strong predictive ability of the models for unknown samples [28].

The OPLS-DA model built to differentiate between rosemary and eucalyptus honey (Figure 2A) comprised one predictive component and four orthogonal components. Reliable discrimination between these honey types was achieved along predictive component 1. Similarly, the rosemary vs. orange blossom honey OPLS-DA model, which was formed by one predictive component and five orthogonal components, showed two clusters of honey samples, but a wider metabolomic composition could be observed within rosemary samples compared to the tight cluster formed by commercially available orange blossom honey (Figure 2B). In addition, good differentiation of orange blossom vs. eucalyptus honey was achieved by the corresponding OPLS-DA model formed by one predictive component and two orthogonal components (Figure 2C).

Permutation tests (200 permutations) confirmed model validity by ensuring that permuted R^2^Y and Q^2^ values were consistently lower than those obtained from the seven-fold CV, with Q^2^ intercepts below 0.05 (Table S2) [28]. Following the confirmation of the high quality of the proposed OPLS-DA models, external sets of honey samples (prediction sets) not used in model construction were used for external validation. The model’s high predictability (Q^2^ = 0.868–0.952) was affirmed by the corresponding misclassification tables, which showed a perfect classification rate (CCR = 100%, Table S3).

This supervised analysis demonstrated that UHPLC-HRMS-based honey fingerprints have significant potential for distinguishing the samples based on their botanical origin. Therefore, these three OPLS-DA models were further analyzed to identify the most distinctive eucalyptus, rosemary, and orange blossom honey metabolites, highlighting their use as potential markers to ensure product traceability.

#### 3.2.2. Experiment 2: OPLS-DA to Discriminate Multifloral and Monofloral Honey

An OPLS-DA model was created to differentiate between multifloral and monofloral honey for the subsequent identification of key monofloral and multifloral markers. Similar to the previous experiment, for model construction, 80% of the observations (96 in total) were used as the training set, while the remaining 20% (24 observations) were randomly chosen for external validation. Detailed validation and performance of the proposed OPLS-DA model are available in Table S2.

**Figure 3:**
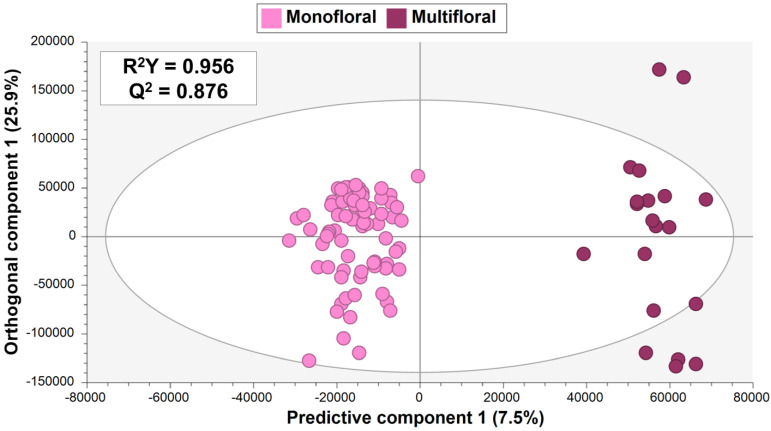
OPLS-DA score plot made of 80% of total honey fingerprints (training set) showing the discrimination between multifloral and monofloral honey samples.

The resulting OPLS-DA model (Figure 3) successfully fulfilled internal CV, including high goodness of fit (R^2^Y = 0.956) and acceptable predictability (Q^2^ = 0.876), along with a significant CV-ANOVA *p*-value of 3.2 × 10^−29^ (Table S2). According to the OPLS-DA score plot, distinguishing multifloral from monofloral honey (Figure 3) included one predictive component and seven orthogonal components. Similarly to PCA findings, the model revealed a wider distribution of multifloral samples compared with eucalyptus, rosemary, and orange blossom honey included within the monofloral sample group.

Permutation tests (200 permutations) validated the model by showing that permuted R^2^Y and Q^2^ values were consistently lower than those from the seven-fold CV, with Q^2^ intercepts (−0.803; −0.805) below 0.05 (Table S2). Moreover, an external set of honey samples (prediction set) not used in model construction was used for further validation, achieving a perfect classification rate considering the resulting misclassification table (CCR = 100%, Table S3).

Therefore, OPLS-DA-based supervised multivariate statistics confirmed the potential of UHPLC-HRMS fingerprinting to discriminate honey samples, in terms of metabolomic diversity, derived from various botanical origins.

### 3.3. Identification of Honey Markers

In the subsequent phase, since the proposed OPLS-DA models demonstrated effective differentiation between monofloral honey samples, as well as reliable multifloral vs. monofloral honey discrimination, the aim was to identify compounds with high discrimination potential between honey sample groups. To achieve this, the VIP tool from SIMCA^®^ software was used at a VIP score threshold of >1.00 to highlight potential honey markers. Additionally, the selection and identification of markers considered metabolites associated with significant differences between sample groups, with Benjamini–Hochberg-adjusted *p*-values < 0.05 and an FC cut-off of 1.10.

As a result, nine different honey metabolites were identified using UHPLC-Q-Orbitrap-HRMS fingerprinting as discriminant compounds, fulfilling these criteria. These markers were classified as level 2, identified based on MS and MS/MS spectral data, using established LC-MS databases and in silico MS/MS fragmentation tools. For each marker, at least two ions (the precursor ion and one or two MS/MS fragment ions) were matched with theoretical MS and MS/MS data from databases, with mass errors < 5 ppm in all cases, supporting the high accuracy of HRMS-based metabolomics for the search of markers. The markers identified in this research are detailed in Table 1, which includes their retention time (RT), molecular formula, VIP score, *p*-value, and log2(FC) value.

Markers were described as specific discriminating key markers to distinguish between pairs of conditions, namely rosemary vs. eucalyptus honey, rosemary vs. orange blossom honey, orange blossom vs. eucalyptus honey, and multifloral vs. monofloral honey (Table 1). As will be discussed later, several marker metabolites have been previously reported in honey, but this is the first study that proposed them as specific markers to distinguish between honey sample groups assessed in this study. Thus, this research represents the first comprehensive analysis of the impact of the botanical origin on honey samples, focusing on identifying novel markers for honey authentication not addressed so far. Further information regarding the selected markers, including a detailed list of precursor and fragment ions, is available in Table S4. According to the markers displayed in Table 1, supervised OPLS-DA modeling, followed by the VIP approach, revealed the significant influence of the botanical origin on the amino acid profile, with 78% of the annotated honey markers being amino acids and derivatives. Additionally, a carbohydrate and an alkaloid derivative (i.e., raffinose and trigonelline, respectively) were also identified among the honey markers (Table 1).

#### 3.3.1. Markers to Discriminate Monofloral Samples (Eucalyptus, Rosemary, and Orange Blossom Honey)

Regarding rosemary vs. eucalyptus honey discrimination, four amino acid markers were proposed, namely L-proline, L-pyroglutamic acid, *N*-(1-deoxy-1-fructosyl)phenylalanine, and L-phenylalanine (Table 1). Three of these markers were noticed to be up-accumulated in eucalyptus vs. rosemary honey, but L-phenylalanine was highlighted as an enhanced metabolite of rosemary honey. A comprehensive discussion of rosemary vs. eucalyptus honey markers is provided later.

**Figure 4:**
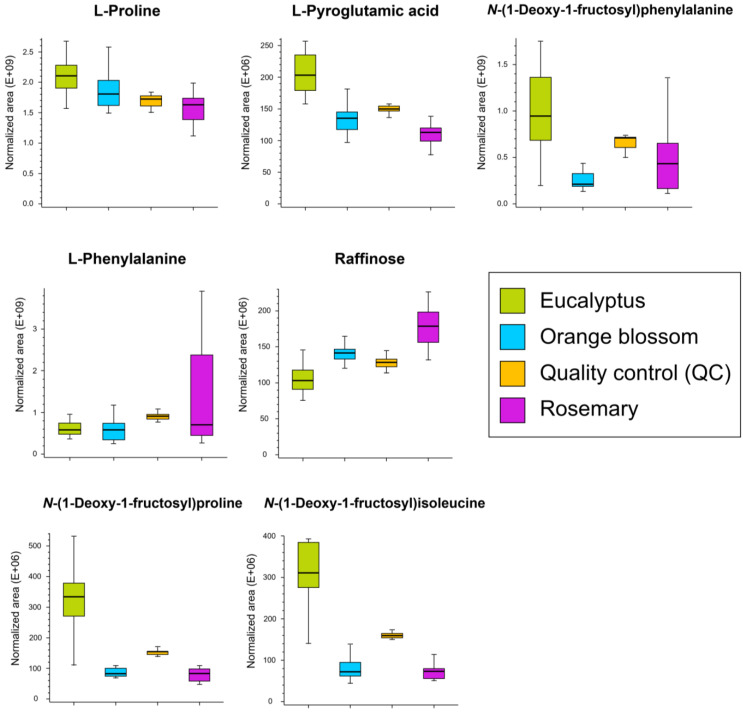
Boxplots built considering normalized peak areas showing the distribution of selected markers (*p*-values < 0.05, VIP scores > 1.00, and FC > 1.10) to discriminate between tested monofloral honey samples (eucalyptus, orange blossom, and rosemary).

L-proline (RT = 1.039 min, VIP score = 2.39, log2(FC) = −0.37 rosemary vs. eucalyptus) was found as a distinctive metabolite down-accumulated in rosemary samples compared to eucalyptus ones. L-proline has been reported within honey’s composition [29] as a product of the salivary secretion of bees during the conversion of nectar into honey [30]. In more detail, L-proline is described as one of the main contributors to the amino acid profile of honey, being the most significant one, comprising >50% of the total amino acids [31]. Moreover, this amino acid constitutes a key compound, bearing in mind that it can help reduce oxidative stress through several mechanisms [32]. In this regard, the statistical findings would reveal eucalyptus honey commercialized within Spanish markets as an important source of L-proline compared to rosemary honey, which has a significantly lower abundance of L-proline (*p*-value = 1.2 × 10^−8^, Table 1), making L-proline an excellent marker to distinguish between this pair of conditions (AUC = 0.927, Figure S3). Corresponding boxplots showing content trends of markers within tested monofloral honey types are displayed in Figure 4.

L-pyroglutamic acid (RT = 1.083 min, VIP score = 1.17, log2(FC) = −0.85 rosemary vs. eucalyptus), also known as 5-oxoproline, is a derivative of L-glutamic acid amino acid, this latter being reported within honey’s chemical composition [2]. The outcomes revealed it as a marker enhanced in eucalyptus honey. Moreover, a maximum AUC value of 1 (Figure S3) was obtained for this marker, evidencing its excellent performance as a key metabolite to discriminate rosemary and eucalyptus honey. L-pyroglutamic acid is also naturally present in different foods, including herbs and spices, such as some of the botanical sources assessed in this study, namely rosemary and *Citrus* plants [29]. L-pyroglutamic acid has been reported as the main amino acid in *Meliponula ferruginea*-derived products (from Tanzania), but in contrast, it is scarcely found in European honeybees from Bulgaria (*A. mellifera*) [33]. In line with these findings, the L-pyroglutamic concentration may vary depending on the type of honey and its origin, highlighting its feasibility as a marker for rosemary vs. eucalyptus honey differentiation.

*N*-(1-deoxy-1-fructosyl)phenylalanine (RT = 1.133 min, VIP score = 2.45) was found as a significant marker of eucalyptus-based honey samples (log2(FC) = −1.12 rosemary vs. eucalyptus, Table 1). It is a well-recognized Amadori compound found in honey [34]. Amadori compounds are early products of the Maillard reaction, which typically occurs during the processing and storage of honey, being precursors of browning and flavor substances that contribute to the sensorial and organoleptic characteristics of honey [35]. The glucose-derived Amadori compounds from amino acids (*N*-1-(deoxy-1-fructosyl)amino acid) are known as fructosyl amino acids [36]. The Maillard reaction, where reducing sugars react with amino acids, may occur even at room temperature as an indicator of honey aging, but it is enhanced by high temperatures [35]. In line with the findings of this study, which revealed the presence of this fructosyl amino acid in eucalyptus and rosemary honey samples, it is worth noting that due to commercial factors, honey may be stored for nearly a year before consumption [37], justifying the overall presence of this fructosyl amino acid in investigated honey samples commercialized in Spain.

In more detail, in honey, the presence of Amadori compounds, including *N*-(1-deoxy-1-fructosyl)phenylalanine, is influenced by several factors, including the honey composition and type, as well as storage and processing conditions. Regarding the first factor, different types of honey may vary in their amino acid and sugar content as a result of their botanical (or floral) source, consequently affecting the extent and rate of the Maillard reaction. Concerning the second factor, storage time plays a key role in the formation of these Maillard intermediates, considering that longer storage times may accelerate the production of Amadori compounds. Consequently, according to the findings of this study, the eucalyptus honey commercialized in Spanish markets contains significantly higher levels of *N*-(1-deoxy-1-fructosyl)phenylalanine compared to the rosemary samples (*p*-value = 2.9 × 10^−3^ and Log2(FC) = −1.12 rosemary vs. eucalyptus, Table 1), revealing the following potential outcomes: (1) eucalyptus honey is darker (compared to rosemary honey), with higher levels of certain amino acids (precursors of the Maillard reaction), which might lead to higher concentrations of Amadori compounds, and (2) eucalyptus samples would have been stored for a longer period, revealing the use of this compound as a potential marker for honey aging. In line with the first point, as previously described, this UHPLC-HRMS untargeted omics study revealed significantly higher levels of L-proline (the main honey amino acid) in eucalyptus honey (Table 1). Additionally, previous research supports the increase in L-proline in honey varieties over storage time [33], which would be in line with higher L-proline levels found in long-storage eucalyptus honey. It is worth mentioning that in line with the higher abundance of *N*-(1-deoxy-1-fructosyl)phenylalanine as an Amadori compound in the assessed eucalyptus samples, these samples were characterized by a darker brown color compared to the slightly yellowish color of rosemary (or orange blossom) samples. In good agreement with this point, Yan et al. explained that some Amadori compounds contribute to the characteristic color of chaste honey [34].

L-phenylalanine (RT = 1.207 min, VIP score = 3.50) was revealed by this untargeted UHPLC-HRMS approach as a significantly up-accumulated marker in rosemary vs. eucalyptus honey (log2(FC) = 0.27 rosemary vs. eucalyptus, Table 1). This amino acid has been reported within honey’s composition [29,30]. Moreover, in good agreement with this outcome, different proteinogenic amino acids, including threonine, alanine, tyrosine, and phenylalanine, have been noticed in rosemary infusions [32]. This marker made evident that the amino acid profile of honey is highly dependent on the pollen species from where the nectar originates, and therefore, it can serve as an indicator for botanical differentiation.

Concerning rosemary vs. orange blossom discrimination, three markers were revealed by OPLS-DA modeling, followed by the VIP approach: L-proline, raffinose, and L-phenylalanine (Table 1). Two of the discriminant markers have been previously outlined as markers of rosemary vs. eucalyptus honey (i.e., L-proline and L-phenylalanine), but raffinose has been newly described as a distinctive marker to differentiate these two kinds of honey. Regarding the aforementioned markers, L-proline (RT = 1.039 min, VIP score = 2.25) was found up-accumulated in orange blossom honey compared to rosemary samples (log2(FC) = −0.15 rosemary vs. orange blossom, Table 1). Significantly higher abundances of L-phenylalanine (RT = 1.207 min, VIP score = 5.55) were found in rosemary honey compared to orange blossom samples (log2(FC) = 0.27 rosemary vs. orange blossom, Table 1). Thus, this untargeted metabolomics approach revealed the potential of L-phenylalanine as a specific enhanced marker of rosemary honey compared to the remaining honey types (eucalyptus and orange blossom).

Raffinose (RT = 1.062 min, VIP score = 1.05, log2(FC) = 0.34 rosemary vs. orange blossom, Table 1) was outlined as a distinctive up-accumulated metabolite in rosemary vs. orange blossom honey, with a good overall marker performance according to the ROC curve (AUC = 0.872, Figure S3). Raffinose is a trisaccharide composed of galactose, fructose, and glucose reported within honey’s saccharide composition [2,29]. According to previous research, it has been highlighted as a potential marker to differentiate between Italian honey varieties (e.g., acacia, chestnut, honeydew, multifloral) based on their botanical origin, being reported as a key component of the honeydew type [38]. In line with these findings, the use of raffinose as a key marker of rosemary samples was postulated for the first time in this study, demonstrating the importance of classifying the botanical origins of honey not studied so far based on effective and key honey components. Moreover, according to the boxplots displayed in Figure 4, the raffinose content was in the following order of abundance within the assessed honey groups: rosemary > orange blossom > eucalyptus. Previous research has identified a negative correlation between enhanced levels of hydroxymethylfurfural (HMF) found when storage time increases and lower abundances of saccharide content, including sucrose, glucose, and raffinose [11]. According to these outcomes, lower raffinose levels in eucalyptus samples highlighted by the UHPLC-HRMS approach would reveal longer storage times. In line with this outcome, this study showed eucalyptus as the honey type characterized by significantly higher abundances of some Amadori compounds as potential early Maillard products (and lower levels of raffinose) resulting from the storage and aging impact (Figure 4).

Four key honey components were identified for the discrimination between orange blossom and eucalyptus honey: L-proline, *N*-(1-deoxy-1-fructosyl)proline, L-pyroglutamic acid, and *N*-(1-deoxy-1-fructosyl)isoleucine (Table 1). All these honey metabolites were highlighted as key and suitable discriminant compounds according to their AUC values ranging from 0.970 to 1 (Figure S3). L-proline presented an AUC value of 0.774 (Figure S3).

L-proline (RT = 1.039 min, VIP score = 1.36, log2(FC) = −0.22 orange blossom vs. eucalyptus) was highlighted as a common marker able to discriminate between the three monofloral honey samples, following this content trend: eucalyptus > orange blossom > rosemary (Figure 4). Thus, it is postulated as a reliable marker of eucalyptus honey compared to the remaining honey types. In good agreement with the results of this study, previous research has supported the up-accumulation of L-proline in eucalyptus samples compared to the *Citrus*-based honey produced in Morocco [31] and South Italia [39].

L-pyroglutamic acid, which has been previously described in this UHPLC-HRMS study as a marker of rosemary vs. eucalyptus honey (log2(FC) = −0.85 rosemary vs. eucalyptus, Table 1), was also outlined as a key component to differentiate between another pair of conditions, namely orange blossom vs. eucalyptus (VIP score = 1.19, log2(FC) = −0.59, Table 1). Thus, these results revealed the same down-accumulation of L-pyroglutamic acid in rosemary and orange blossom honey when compared to the eucalyptus type.

The remaining two markers to distinguish between orange blossom and eucalyptus honey were identified as two Amadori compounds: *N*-(1-deoxy-1-fructosyl)proline and *N*-(1-deoxy-1-fructosyl)isoleucine (Table 1). Both fructosyl amino acids were found in significantly higher levels in eucalyptus samples (log2(FC) values of −2.02 and −2.10, Table 1). Accordingly, these two Amadori compounds followed the same concentration trend as the one described for *N*-(1-deoxy-1-fructosyl)phenylalanine, a key enhanced compound in eucalyptus samples compared to rosemary (Table 1). Particularly, *N*-(1-deoxy-1-fructosyl)proline was found in the eucalyptus, orange blossom, and rosemary samples of this study, in line with its previously reported occurrence in the long-term storage of chaste honey [34]. Interestingly, *N*-(1-deoxy-1-fructosyl)isoleucine was described for the first time in honey samples as an Amadori compound.

**Figure 5:**
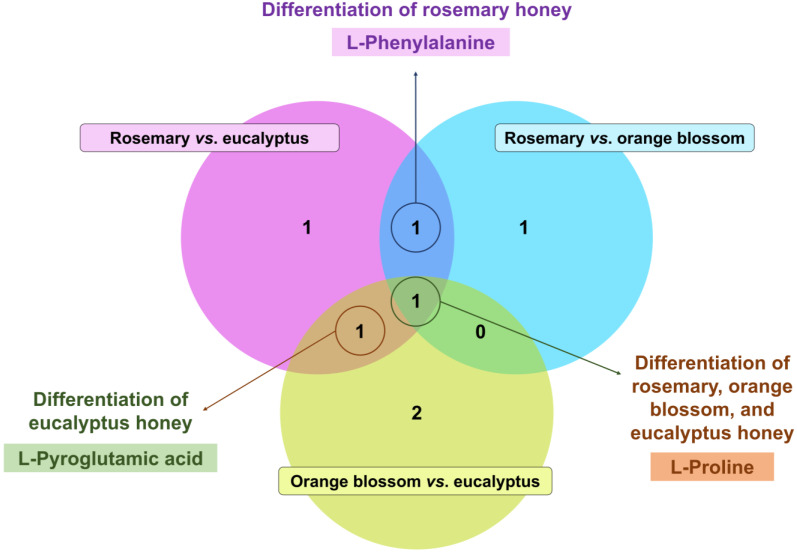
Venn diagram built considering significant markers (*p*-values < 0.05, VIP scores > 1.00, and FC > 1.10) to discriminate tested monofloral honey samples. The following pairwise comparisons were performed: rosemary vs. eucalyptus honey, rosemary vs. orange blossom honey, and orange blossom vs. eucalyptus honey.

Finally, bearing in mind that some markers were common between pairs of conditions, a Venn diagram was created (Figure 5) for an overview of their significance and overlapping to distinguish between the assessed monofloral honey sample groups. As displayed in Figure 5, only one marker, i.e., L-proline, served as a significant metabolite to discriminate between the three types of assessed honey. Rosemary vs. eucalyptus and rosemary vs. orange blossom markers revealed L-phenylalanine as a significant up-accumulated metabolite distinctive of rosemary samples. Similarly, the Venn diagram findings suggested the potential use of L-pyroglutamic acid as a key enhanced component of eucalyptus samples compared to the remaining botanical sources.

#### 3.3.2. Markers to Discriminate Multifloral vs. Monofloral Honey

**Figure 6:**
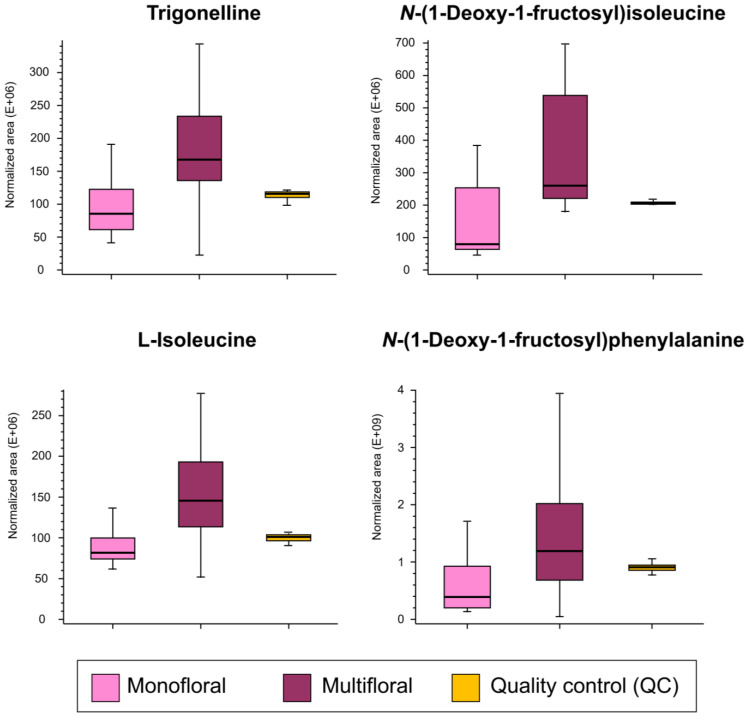
Boxplots built considering normalized peak areas showing the distribution of selected markers (*p*-values < 0.05, VIP scores > 1.00, and FC > 1.10) to discriminate between multifloral and monofloral honey samples (eucalyptus, orange blossom, and rosemary honey).

The second goal of this study was to assess the overall metabolomic differences between multifloral and monofloral (eucalyptus, rosemary, and orange blossom types) honey. OPLS-DA modeling (Figure 3), followed by the VIP approach, revealed the feasibility of four markers to effectively distinguish between multifloral and monofloral honey: trigonelline, *N*-(1-deoxy-1-fructosyl)isoleucine, L-isoleucine, and *N*-(1-deoxy-1-fructosyl)phenylalanine (Table 1). *N*-(1-deoxy-1-fructosyl)isoleucine and *N*-(1-deoxy-1-fructosyl)phenylalanine have been previously discussed as markers to discriminate between monofloral samples, whereas trigonelline and L-isoleucine were reported as new markers to achieve multifloral vs. monofloral differentiation. Interestingly, these four potential multifloral vs. monofloral markers followed the same concentration trend since they were up-accumulated in multifloral samples compared to monofloral honey (positive log2(FC) values ranging from 0.83 to 1.70 were obtained; Table 1). Boxplots of selected markers to discriminate multifloral vs. monofloral honey samples are shown in Figure 6. It is worth mentioning that all the selected VIP markers presented acceptable AUC values from 0.763 to 0.831 (Figure S4).

Trigonelline (RT = 1.035 min, VIP score = 1.45, log2(FC) = 0.97 multifloral vs. monofloral) is an alkaloid reported within herbs and spices, such as alfalfa, fennel, and parsley [29], this fact being in line with the different potential botanical sources of multifloral-labeled honey samples. In more detail, trigonelline is found not only in these botanical sources but also in Chinese honey from different botanical origins [11]. In line with the findings of this study that proposed trigonelline as a potential marker for multifloral vs. monofloral differentiation, Wang et al. developed a targeted methodology based on the determination of trigonelline to identify honey’s botanical origins (i.e., rape, *Citrus*, and coffee honey). In this particular case, trigonelline was found up-accumulated in rape honey [40].

Regarding Amadori compounds, namely *N*-(1-deoxy-1-fructosyl)isoleucine (RT = 1.089 min, VIP score = 2.49, log2(FC) = 1.70 multifloral vs. monofloral) and *N*-(1-deoxy-1-fructosyl)phenylalanine (RT = 1.133 min, VIP score = 4.88, log2(FC) = 1.62 multifloral vs. monofloral), both were key marker compounds found to be enhanced in multifloral samples compared to eucalyptus, rosemary, and orange blossom samples, englobed within the monofloral sample group (Table 1). At this point, it is worth noting that the Maillard reaction depends on the availability of reducing sugars and amino acids. Bearing in mind the higher diversity of nectar sources in multifloral honey samples, they might have a higher availability of both, enhancing the Maillard reaction and resulting in higher concentrations of these compounds. In contrast, bees producing monofloral honey are limited to specific plant species, which might not have as high levels of the amino acids that form *N*-(1-deoxy-1-fructosyl) derivatives. Additionally, processing factors, such as storage time, could also play a significant role in these differences within sample groups.

Finally, in line with the potential higher availability of amino acids in honey varieties as a result of their broader range of botanical sources, OPLS-DA and VIP highlighted L-isoleucine (RT = 1.102 min, VIP score = 1.42, log2(FC) = 0.83 multifloral vs. monofloral) as a key marker found in significantly higher levels in multifloral vs. monofloral honey samples (*p*-value = 3.9 × 10^−4^, Table 1). Previous literature has supported the occurrence of L-isoleucine in honey [30,39].

Thus, the results of this study indicate that the botanical origin significantly influences the final metabolomic composition of honey. This study pioneered exploring the specific markers resulting from the impact of the botanical origin on the metabolomic profile of honey, highlighting for the first time the specific use of key marker honey compounds to distinguish between investigated sample groups that are widely commercialized in Spanish markets.

## 4. Conclusions

This study assessed the metabolomic differences arising from different botanical origins in a wide range of honey varieties commercialized in Spanish markets, specifically eucalyptus, rosemary, orange blossom, and multifloral types. The identification of specific honey markers to discriminate between these groups, a topic not previously addressed in the literature, was performed using a novel UHPLC-Q-Orbitrap-HRMS method. The HCA/PCA models indicated significant variability in multifloral honey, while OPLS-DA models showed strong discrimination power to highlight metabolomic differences between multifloral and monofloral honey. Specific markers of eucalyptus, rosemary, and orange blossom honey were identified, enhancing honey traceability. Key amino acid and derivative markers—L-proline, L-pyroglutamic acid, *N*-(1-deoxy-1-fructosyl)phenylalanine, L-phenylalanine, *N*-(1-deoxy-1-fructosyl)proline, and *N*-(1-deoxy-1-fructosyl)isoleucine—and one trisaccharide (raffinose) were identified for monofloral samples. Notably, *N*-(1-deoxy-1-fructosyl)amino acids (Amadori compounds) were found in higher concentrations in eucalyptus honey, suggesting that they may serve as markers for honey aging and botanical origin differentiation. The study also identified significant markers of rosemary honey (L-phenylalanine and raffinose) and multifloral honey (trigonelline; L-isoleucine; and two Amadori compounds, namely *N*-(1-deoxy-1-fructosyl)isoleucine and *N*-(1-deoxy-1-fructosyl)phenylalanine). These markers were up-accumulated in multifloral samples, indicating a potential greater availability of reducing sugars and amino acids in multifloral honey, enhancing the Maillard reaction. This research highlights the significant influence of the botanical origin on honey’s metabolomic composition, offering novel markers for the differentiation of commercial honey varieties by effective UHPLC-HRMS-based metabolomics.

## Figures and Tables

**Table 1:** List of markers to distinguish between honey sample groups. Metabolites were selected by UHPLC-Q-Orbitrap-HRMS fingerprinting combined with OPLS-DA modeling, followed by the VIP approach (VIP score threshold > 1.00, significant *p*-values < 0.05, and FC cut-off of 1.10).

No.	RT (min)	Marker Name	Molecular Formula	VIP Score	*p*-Value ^1^	Log2(FC) ^2^
**Rosemary vs. eucalyptus honey**						
1	1.039	L-proline	C_5_H_9_NO_2_	2.39 ± 1.06	1.2 × 10^−8^	−0.37
2	1.083	L-pyroglutamic acid	C_5_H_7_NO_3_	1.17 ± 0.74	8.9 × 10^−9^	−0.85
3	1.133	*N*-(1-deoxy-1-fructosyl)phenylalanine	C_15_H_21_NO_7_	2.45 ± 0.71	2.9 × 10^−3^	−1.12
4	1.207	L-phenylalanine	C_9_H_12_NO_2_	3.50 ± 1.88	2.9 × 10^−3^	0.27
**Rosemary vs. orange blossom honey**						
5	1.039	L-proline	C_5_H_9_NO_2_	2.25 ± 1.36	2.8 × 10^−2^	−0.15
6	1.062	Raffinose	C_18_H_32_0_16_	1.05 ± 0.55	9.4 × 10^−7^	0.34
7	1.207	L-phenylalanine	C_9_H_12_NO_2_	5.55 ± 2.08	5.0 × 10^−3^	0.27
**Orange blossom vs. eucalyptus honey**						
8	1.039	L-proline	C_5_H_9_NO_2_	1.36 ± 0.66	3.7 × 10^−3^	−0.22
9	1.040	*N*-(1-deoxy-1-fructosyl)proline	C_11_H_19_NO_7_	1.97 ± 0.30	8.9 × 10^−9^	−2.02
10	1.083	L-pyroglutamic acid	C_5_H_7_NO_3_	1.19 ± 0.80	1.8 × 10^−7^	−0.59
11	1.089	*N*-(1-deoxy-1-fructosyl)isoleucine	C_12_H_23_NO_7_	1.95 ± 0.52	8.7 × 10^−9^	−2.10
**Multifloral vs. monofloral honey**						
12	1.035	Trigonelline	C_7_H_7_NO_2_	1.45 ± 0.34	1.4 × 10^−3^	0.97
13	1.089	*N*-(1-deoxy-1-fructosyl)isoleucine	C_12_H_23_NO_7_	2.49 ± 1.40	6.3 × 10^−5^	1.70
14	1.102	L-isoleucine	C_6_H_13_NO_2_	1.42 ± 0.57	3.9 × 10^−4^	0.83
15	1.133	*N*-(1-deoxy-1-fructosyl)phenylalanine	C_15_H_21_NO_7_	4.88 ± 1.12	7.3 × 10^−3^	1.62

^1^ *p*-Values calculated by one-way ANOVA with Tukey’s post-hoc test for eucalyptus, rosemary, and orange blossom honey comparison groups. Two-tailed Student’s *t*-test was used for multifloral vs. monofloral honey comparison. All *p*-values were adjusted using Benjamini–Hochberg correction for the false discovery rate. ^2^ Results of fold change (FC) analysis performed for the comparison of honey samples (FC cut-off of 1.10) expressed as log2(FC) values.

## Data Availability

The original contributions presented in the study are included in the article/Supplementary Materials, further inquiries can be directed to the corresponding author.

## Supplementary Materials

The following supporting information can be downloaded at https://www.mdpi.com/article/10.3390/foods13172755/s1, Figure S1: Representative total ion chromatograms (TICs) of multifloral and monofloral honey samples obtained by the full-scan acquisition mode in ESI+ and ESI− polarities by untargeted UHPLC-Q-Orbitrap-HRMS analysis: (A,B) multifloral, (C,D) eucalyptus, (E,F) rosemary, and (G,H) orange blossom honey; Figure S2: Control chart obtained by monitoring the chromatographic peak area of the procedure internal standard (chlorantraniliprole) added to each honey sample during sample preparation. The upper control limit (UCL) and the lower control limit (LCL) (±3 × standard deviation) and average peak area values are indicated; Figure S3: Receiver operating characteristics (ROC) curve and the area under the ROC curve (AUC) evaluated for the specific performance of each proposed marker to distinguish between monofloral honey samples; Figure S4: Receiver operating characteristics (ROC) curve and the area under the ROC curve (AUC) evaluated for the specific performance of each proposed marker to distinguish between multifloral and monofloral honey samples; Table S1: List of honey samples investigated in this study to select key markers to distinguish between monofloral samples (eucalyptus, rosemary, and orange blossom honey) and monofloral vs. multifloral honey; Table S2: Performance and validation parameters for the supervised OPLS-DA models built for the botanical origin discrimination of honey samples; Table S3: Misclassification table obtained for the blind prediction of samples of the prediction set that were not considered for OPLS-DA model building; Table S4: Additional information about selected marker honey metabolites.
