# Supplementary material for: Identifying Key Markers for Monofloral (Eucalyptus, Rosemary, and Orange Blossom) and Multifloral Honey Differentiation in the Spanish Market by UHPLC-Q-Orbitrap-High-Resolution Mass Spectrometry Fingerprinting and Chemometrics
Source: Foods. 2024 Aug 29;13(17):2755. doi: 10.3390/foods13172755 (PMC11395089; doi:10.3390/foods13172755)
Supplement: Supplementary file 1 [file foods-13-02755-s001.zip › foods-3160639-supplementary.pdf]

Journal name: Foods

SUPPLEMENTARY DATA to the paper entitled:

Identifying Key Markers for Monofloral (Eucalyptus, Rosemary, and Orange Blossom) and Multifloral Honey Differentiation in the Spanish Market by UHPLC-Q-Resolution Mass Spectrometry Fingerprinting and Chemometrics

Araceli Rivera-Pérez, Alba María Navarro-Herrera and Antonia Garrido Frenich *

Research Group "Analytical Chemistry of Contaminants", Department of Chemistry and Physics, Research Centre for Mediterranean Intensive Agrosystems and Agrifood Biotechnology (CIAIMBITAL), Agrifood Campus of International Excellence (ceiA3), University of Almeria, E-04120 Almeria, Spain

* Correspondence: agarrido@ual.es

ORCID CODES AND E-MAIL ADDRESS

Araceli Rivera-Pérez: 0000-0003-1099-7185 (arp800@ual.es)

Antonia Garrido Frenich: 0000-0002-7904-7842 (agarrido@ual.es)

Supplementary contents

Supplementary tables

Table S1. List of honey samples investigated in this study to select key markers to distinguish between monofloral samples (eucalyptus, rosemary, and orange blossom honey) and monofloral *vs*. multifloral honey.

Table S2. Performance and validation parameters for the supervised OPLS-DA models built for the botanical origin discrimination of honey samples.

Table S3. Misclassification table obtained for the blind prediction of samples of the prediction set that were not considered for OPLS-DA model building.

Table S4. Additional information about selected marker honey metabolites.

Supplementary figures

Figure S1. Representative total ion chromatograms (TICs) of multifloral and monofloral honey samples obtained by the full-scan acquisition mode in ESI+ and ESI− polarities by untargeted UHPLC-Q-Orbitrap-HRMS analysis: (A,B) multifloral, (C,D) eucalyptus, (E,F) rosemary, and (G,H) orange blossom honey.

Figure S2. Control chart obtained by monitoring the chromatographic peak area of the procedure internal standard (chlorantraniliprole) added to each honey sample during sample preparation. The upper control limit (UCL) and the lower control limit (LCL) (±3 × standard deviation) and average peak area values are indicated.

Figure S3. Receiver operating characteristics (ROC) curve and the area under the ROC curve (AUC) evaluated for the specific performance of each proposed marker to distinguish between monofloral honey samples.

Figure S4. Receiver operating characteristics (ROC) curve and the area under the ROC curve (AUC) evaluated for the specific performance of each proposed marker to distinguish between multifloral and monofloral honey samples.

Table S1. List of honey samples investigated in this study to select key markers to distinguish between monofloral samples (eucalyptus, rosemary, and orange blossom honey) and monofloral *vs*. multifloral honey.

No.	Honey type	Botanical origin	Geographical origin	Commercial brand
**1**	Monofloral	Eucalyptus	Spain, Argentina	Auchan
**2**	Monofloral	Eucalyptus	Uruguay	Luna de miel
**3**	Monofloral	Eucalyptus	Spain, Uruguay	Consum
**4**	Monofloral	Eucalyptus	Spain	Corte Inglés
**5**	Monofloral	Eucalyptus	Spain, Uruguay	Lidl
**6**	Monofloral	Eucalyptus	Spain, Uruguay	Carrefour
**7**	Monofloral	Eucalyptus	Spain, Argentina	Hacendado
**8**	Monofloral	Eucalyptus	Spain	Mellarius
**9**	Monofloral	Eucalyptus	Spain	Mielove
**10**	Monofloral	Eucalyptus	Spain, Uruguay, Argentina	Aldi
**11**	Monofloral	Rosemary	Spain	Auchan
**12**	Monofloral	Rosemary	Spain	Brezal
**13**	Monofloral	Rosemary	Spain	Luna de miel
**14**	Monofloral	Rosemary	Spain	Miel de Alcarria
**15**	Monofloral	Rosemary	Spain	Sierra de Gádor
**16**	Monofloral	Rosemary	Spain	Consum
**17**	Monofloral	Rosemary	Spain	Helios
**18**	Monofloral	Rosemary	Spain	Lidl
**19**	Monofloral	Rosemary	Spain	Carrefour
**20**	Monofloral	Rosemary	Spain	Hacendado
**21**	Monofloral	Orange blossom	Spain	Auchan
**22**	Monofloral	Orange blossom	Spain	Luna de miel
**23**	Monofloral	Orange blossom	Spain	Consum
**24**	Monofloral	Orange blossom	Spain	Corte Inglés
**25**	Monofloral	Orange blossom	Spain	Helios
**26**	Monofloral	Orange blossom	Spain	Lidl
**27**	Monofloral	Orange blossom	Spain	Mielove
**28**	Monofloral	Orange blossom	Spain	Carrefour
**29**	Monofloral	Orange blossom	Spain	Ifa

**30**	Monofloral	Orange blossom	Spain	Aldi
**31**	Multifloral	Multifloral	EU, Non-EU	Lidl
**32**	Multifloral	Multifloral	Spain	Miel de Alcarria
**33**	Multifloral	Multifloral	Spain	Sierra de Gádor
**34**	Multifloral	Multifloral	Spain, Argentina, Cuba	Consum
**35**	Multifloral	Multifloral	Spain	Helios
**36**	Multifloral	Multifloral	Spain, Turkey, Mexico, El Salvador, Uruguay	Mielove
**37**	Multifloral	Multifloral	China	Didilo
**38**	Multifloral	Multifloral	Spain, Uruguay, China	Alteza
**39**	Multifloral	Multifloral	Spain, Ukraine, Bulgaria, Romania, Argentina, Uruguay	Granja San Francisco
**40**	Multifloral	Multifloral	Spain, Ukraine, China, Argentina, Vietnam	Gourmet

Table S2. Performance and validation parameters for the supervised OPLS-DA models built for the botanical origin discrimination of honey samples.

Model parameters	Rosemary *vs*. eucalyptus	Rosemary *vs*. orange blossom	Orange blossom *vs*. eucalyptus	Multifloral *vs*. monofloral
**R^2X**	0.689	0.671	0.559	0.786
**R^2Y**	0.981	0.973	0.929	0.956
**Q^2**	0.952	0.868	0.895	0.876
**NC[a]**	1 + 4	1 + 5	1 + 2	1 + 7
**Permutation tests (R^2; Q^2 intercepts)**	0.491; -0.873 (rosemary)<br>0.495; -0.815 (eucalyptus)	0.693; -1.020 (rosemary)<br>0.690; -0.982 (orange blossom)	0.316; -0.632 (orange blossom)<br>0.332; -0.594 (eucalyptus)	0.521; -0.803 (multifloral)<br>0.523; -0.805 (monofloral)
**CV-ANOVA *p*-value**	< 0.05 (2.9E-21)	< 0.05 (6.1E-12)	< 0.05 (1.8E-18)	< 0.05 (3.2E-29)
**Fisher's probability**	1.1E-3	1.1E-3	1.1E-3	5.1E-7
**CCR (%)[b]**	100	100	100	100

[a] NC: No. of components expressed as "No. of predictive components + No. of orthogonal components" for OPLS-DA.
[b] CCR: Correct classification rate of prediction samples indicated as a percentage (%).

Table S3. Misclassification table obtained for the blind prediction of samples of the prediction set that were not considered for OPLS-DA model building.

**Rosemary *vs.* eucalyptus**	**Members**	**Correct**	**Eucalyptus**	**Rosemary**
**Eucalyptus**	6	100%	6	0
**Rosemary**	6	100%	0	6
**No class**	0		0	0
Total	12	**100%**	6	6
Fisher's prob.	0.0011			
**Rosemary *vs.* orange blossom**	**Members**	**Correct**	**Orange blossom**	**Rosemary**
**Orange blossom**	6	100%	6	0
**Rosemary**	6	100%	0	6
**No class**	0		0	0
Total	12	**100%**	6	6
Fisher's prob.	0.0011			
**Orange blossom *vs.* eucalyptus**	**Members**	**Correct**	**Eucalyptus**	**Orange blossom**
**Eucalyptus**	6	100%	6	0
**Orange blossom**	6	100%	0	6
**No class**	0		0	0
Total	12	**100%**	6	6
Fisher's prob.	0.0011			
**Multifloral *vs.* monofloral**	**Members**	**Correct**	**Monofloral**	**Multifloral**
**Monofloral**	14	100%	14	0
**Multifloral**	10	100%	0	10
**No class**	0		0	0
Total	24	**100%**	14	10
Fisher's prob.	5.1E-07			

Table S4. Additional information about selected marker honey metabolites.

No.	RT (min)	Marker name	Molecular formula	Assigned adduct	Precursor ion exact mass (*m/z*)[a]	MS/MS fragment ion exact mass (*m/z*)[a]
**1**	1.035	Trigonelline	$C_7H_7NO_2$	$[M+H]^+$	138.05496 ($C_7H_8NO_2$)	110.06004 (C_6H_8NO)<br>94.06513 (C_6H_8N)
**2**	1.039	L-Proline	$C_5H_9NO_2$	$[M+H]^+$	116.07061 ($C_5H_{10}NO_2$)	70.06513 (C_4H_8N)
**3**	1.040	*N*-(1-Deoxy-1-fructosyl)proline	$C_{11}H_{19}NO_7$	$[M+H]^+$	278.12342 ($C_{11}H_{20}NO_7$)	260.11286 ($C_{11}H_{18}NO_6$)<br>242.10229 ($C_{11}H_{16}NO_5$)
**4**	1.062	Raffinose	$C_{18}H_{32}O_{16}$	$[M-H]^-$	503.16176 ($C_{18}H_{31}O_{16}$)	221.06670 ($C_8H_{13}O_7$)<br>161.04555 ($C_6H_9O_5$)
**5**	1.083	L-Pyroglutamic acid	$C_5H_7NO_3$	$[M+H]^+$	130.04987 ($C_5H_8NO_3$)	84.04439 ($C_4H_6NO_3$)
**6**	1.089	*N*-(1-Deoxy-1-fructosyl)isoleucine	$C_{12}H_{23}NO_7$	$[M+H]^+$	294.15472 ($C_{12}H_{24}NO_7$)	276.14416 ($C_{12}H_{22}NO_6$)<br>258.13359 ($C_{12}H_{20}NO_5$)
**7**	1.102	L-Isoleucine	$C_6H_{13}NO_2$	$[M+H]^+$	132.10191 ($C_6H_{14}NO_2$)	86.09643 ($C_5H_{12}N$)<br>69.06988 (C_5H_9)
**8**	1.133	*N*-(1-Deoxy-1-fructosyl)phenylalanine	$C_{15}H_{21}NO_7$	$[M+H]^+$	328.13907 ($C_{15}H_{22}NO_7$)	310.12851 ($C_{15}H_{20}NO_6$)<br>282.13360 ($C_{14}H_{20}NO_5$)
**9**	1.207	L-Phenylalanine	$C_9H_{11}NO_2$	$[M+H]^+$	166.08626 ($C_9H_{12}NO_2$)	120.08078 ($C_8H_{10}N$)<br>103.05423 (C_8H_7)

[a] Theoretical MS and MS/MS ions retrieved from Mass Frontier™ software, CFM-ID, MetFrag, and MS/MS databases such as mzCloud detected in honey samples with mass error < 5 ppm.

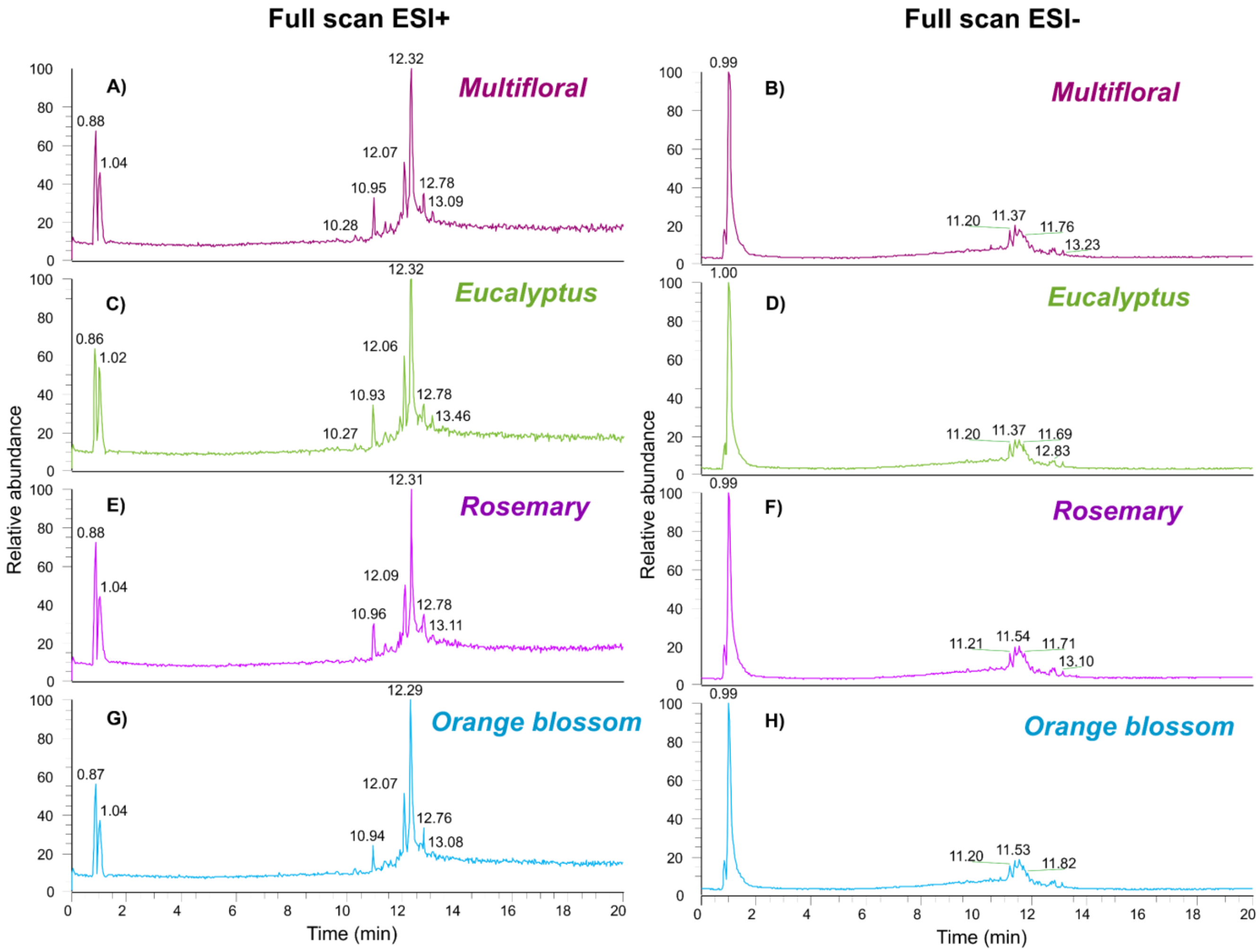

Figure S1. Representative total ion chromatograms (TICs) of multifloral and monofloral honey samples obtained by the full-scan acquisition mode in ESI+ and ESI− polarities by untargeted UHPLC-Q-Orbitrap-HRMS analysis: (A,B) multifloral, (C,D) eucalyptus, (E,F) rosemary, and (G,H) orange blossom honey.

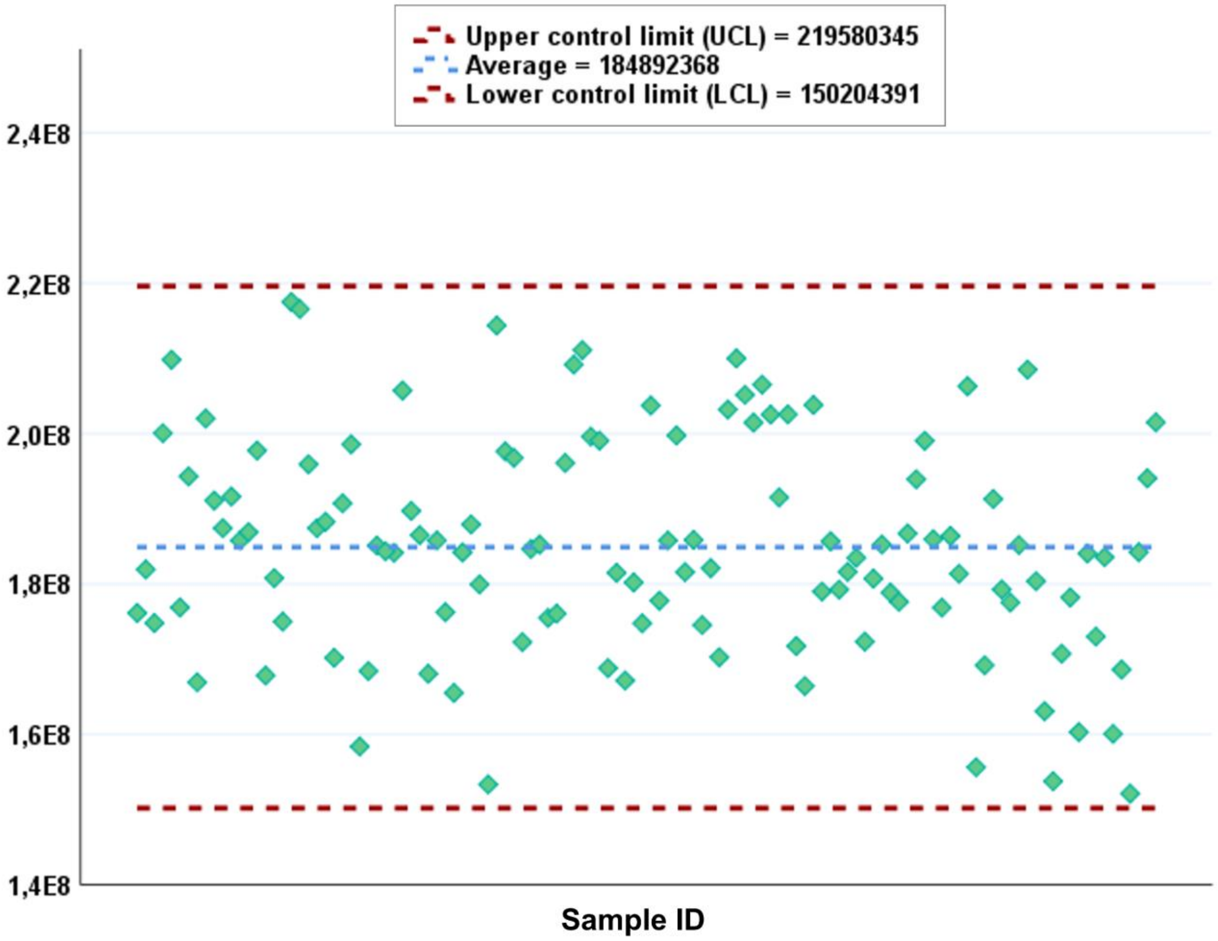

Figure S2. Control chart obtained by monitoring the chromatographic peak area of the procedure internal standard (chlorantraniliprole) added to each honey sample during sample preparation. The upper control limit (UCL) and lower control limit (LCL) (±3 × standard deviation) and average peak area values are indicated.

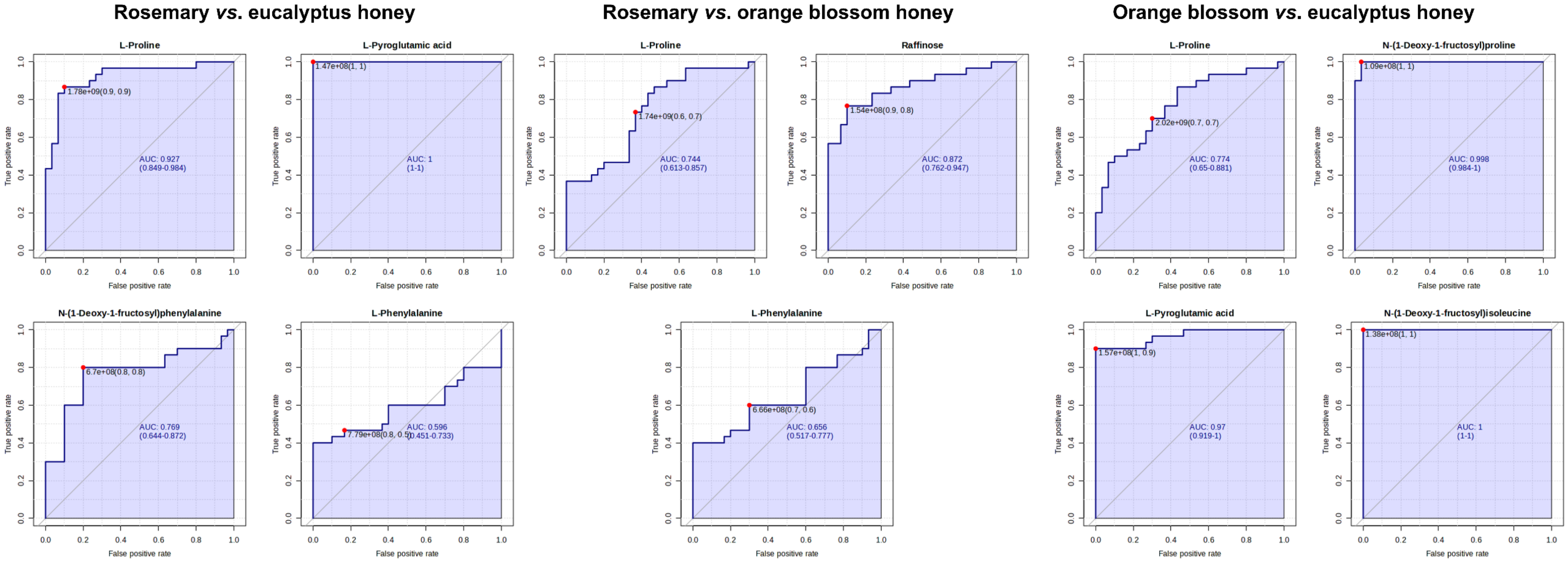

Figure S3. Receiver operating characteristics (ROC) curve and the area under the ROC curve (AUC) evaluated for the specific performance of each proposed marker to distinguish between monofloral honey samples.

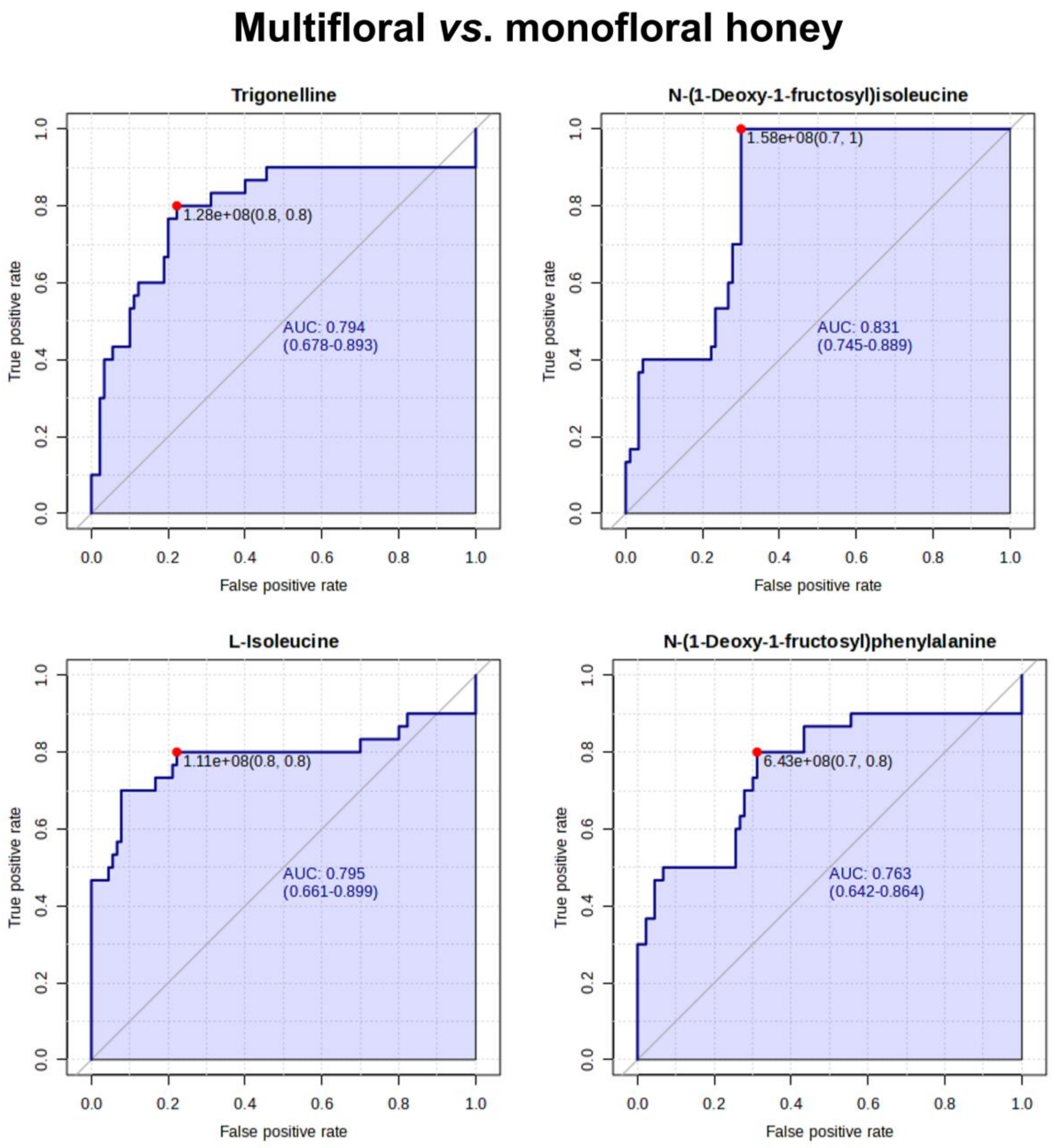

Figure S4. Receiver operating characteristics (ROC) curve and the area under the ROC curve (AUC) evaluated for the specific performance of each proposed marker to distinguish between multifloral and monofloral honey samples.
